# Effect of being a persistent picky eater on feeding difficulties in school-aged children

**DOI:** 10.1016/j.appet.2023.106483

**Published:** 2023-02-03

**Authors:** Dimitrios V. Diamantis, Pauline M. Emmett, Caroline M. Taylor

**Affiliations:** a Bristol Medical School, University of Bristol, Bristol, UK; b Centre for Academic Child Health, Bristol Medical School, University of Bristol, Bristol, UK

**Keywords:** Picky eating, Persistent picky eating, Feeding difficulties, School-aged children, ALSPAC, Fussy eating, Choosy eating

## Abstract

Children who are picky eaters often develop feeding difficulties during preschool years. These difficulties may persist into adolescence in some children. The study aim was to examine feeding difficulties and maternal feeding strategies longitudinally from age 5.5–8.5 years in relation to persistent picky eating. Picky eating behaviour in children enrolled in the Avon Longitudinal Study of Parents and Children was assessed using questionnaires between 2 and 5.5 years of age. Feeding behaviours were evaluated using questionnaires between 5.5 and 8.5 years. Data were analysed using adjusted logistic regression models. Of the 7405 children with data on picky eating 1926 (26%) were classified as never picky eaters, 385 (5%) were non-persistent picky eaters and 564 (8%) were persistent picky eaters. At 5.5, 7 and 8.5 years both persistent picky eaters and non-persistent picky eaters were more likely than never picky eaters to indicate difficulties in eating what the mother wanted, deliberately eat insufficiently, refuse to eat what was on offer, be choosy, not over-eat, and be difficult to get into routine, but with the likelihood decreasing with age (e.g. in persistent picky eaters vs non-picky eaters: refused to eat offered food OR 44.2 (95% CI 29.1, 67.0) at 5.5 years, 15.5 (11.5, 20.8) at 7 years and 14.1 (10.7, 18.6) at 8.5 years). The families of children who are picky eaters at the time of entering the school system should be offered reassurance that the feeding difficulties are likely to slowly resolve over time.

## 1 Introduction

Picky eating is often a source of parental concern, frequently resulting in considerable child and parental distress (Mascola, Bryson, & Agras, 2010). It is generally characterised by fear and unwillingness to try and taste novel foods (food neophobia), together with avoidance of some familiar foods and having strong food preferences (Dovey, Staples, Gibson, & Halford, 2008; Mascola et al., 2010; Taylor, Wernimont, Northstone, & Emmett, 2015). The prevalence of picky eating behaviour varies by identification method (6%–50%), but usually reaches a peak at 3 years old. It is generally regarded as a ‘normal’ phase of development and prevalence substantially decreases at about 5 years old **(**for example, prevalence 15% at age 3 years and 12% at 5 years (Taylor et al., 2015); 28% at age 3 years and 13% at age 6 years (Cardona Cano et al., 2015)). However, the duration of picky eating can vary and can persist into early adolescence (Jacobi, Schmitz, & Agras, 2008; Mascola et al., 2010).

Children who are persistent picky eaters (PPE) should be distinguished from those in whom pickiness resolves in the early years (Non-PPE) as the consequences on growth, development and mental health may differ (Taylor & Emmett, 2019; Tharner et al., 2014). Picky eaters may demand specific meal preparation and/or consume a limited variety of foods (Mascola et al., 2010; Taylor & Emmett, 2019; Tharner et al., 2014): this selective eating can be more evident in PPE and can impact on several psychological and behavioural aspects of the child’s health (Taylor & Emmett, 2019), on developmental difficulties during school years (Cardona Cano et al., 2016; Taylor & Emmett, 2019), and even in some rare cases proceed to eating disorders (Kotler, Cohen, Davies, Pine, & Walsh, 2001; Marchi & Cohen, 1990).

Slow weight gain is a further concern (Brown, 2016). Children with picky eating behaviour tend to have less than the appropriate-for-age daily energy consumption (Volger et al., 2017) and are more likely to be underweight at preschool age (Dubois, Farmer, Girard, Peterson, & Tatone-Tokuda, 2007; Tharner et al., 2014), with potential impact on long-term growth (Taylor & Emmett, 2019). Picky eating behaviour can remain through childhood and adolescence (Levene & Williams, 2018; Mascola et al., 2010), leading in some cases to adult picky eating. This can severely impact social interactions (Kauer, Pelchat, Rozin, & Zickgraf, 2015). Thus, early intervention is important to minimise PPE and its adverse effects.

Children who have picky eating behaviour tend to have difficulties during mealtimes (Dovey et al., 2008; Mascola et al., 2010; Taylor et al., 2015), with parents finding it harder to control the child’s eating patterns (Taylor et al., 2015). Parental worry can result in stressful and harmful practices for the child’s health and lead to the continuation of picky eating (Dovey et al., 2008; Emmett, Hays, & Taylor, 2018; Hafstad, Abebe, Torgersen, & von Soest, 2013; Levene & Williams, 2018; Taylor & Emmett, 2019), as parents will often prioritise short-term adequate food consumption over longer term strategies (Moore, Tapper, & Murphy, 2010). As this worry can be a great source of parental distress, parents of PPE will have substantially more worry overall (Mascola et al., 2010) than parents of children whose pickiness resolves. This may lead to harmful feeding practices, particularly pressure to eat (Berge et al., 2017).

An understanding of the behavioural patterns around food and mealtimes of children with picky eating behaviour is key to understanding problematic feeding behaviours, as well as to broadening understanding for healthcare providers who provide advice to parents. The primary aim of this study was to assess whether PPE in a preschool children (2–5.5 years) predicts feeding difficulties from age 5.5–8.5 years. This comprised: (1) exploring the associations of PPE with feeding behaviours, mealtime patterns, and child and parental perceptions/behaviours around food/meal consumption, including parental strategies to address difficulties; and (2) examining if early maternal worry about feeding is associated with the continuation of adverse feeding behaviours during school age in children with picky eating behaviour.

## 2 Methods

### 2.1 Study design

The Avon Longitudinal Study of Parents and Children (ALSPAC) is an observational population-based prospective longitudinal study, that investigates genetic and environmental influences on children’s health, development, and behaviour (Boyd et al., 2013; Fraser et al., 2013). All pregnant women with expected delivery date between April 1991 and December 1992 who were living in the prescribed area of the former Avon Health Authority were eligible to participate. Further information on the ALSPAC study design, procedures and informed consent can be found at www.bris.ac.uk/alspac. The study website contains details of all the data that are available through a fully searchable data dictionary and variable search tool (http://www.bristol.ac.uk/alspac/researchers/our-data/). Ethics approval was obtained from the ALSPAC Ethics and Law Committee and Local Research Ethics Committees. Informed consent for the use of data collected via questionnaire and clinics was obtained from participants following the recommendations of the ALSPAC Ethics and Law Committee at the time. The analytic plan was pre-specified. Any data-driven analyses are clearly identified and discussed appropriately.

### 2.2 Data collection

The primary data collection took place through self-completed postal questionnaires, available at http://www.bristol.ac.uk/alspac/researchers/questionnaires/. The primary caregiver (mostly the mother) completed questionnaires at various timepoints, starting in pregnancy. The child’s sex and birthweight were obtained through medical records. The remaining information on the caregiver and child’s background and parental demographic factors was obtained through these questionnaires.

### 2.3 Definition and longitudinal classification of picky eating

The following question was asked when the child was 2, 3, 4.5 and 5.5 years old: ‘Does your child have definite likes and dislikes as far as food is concerned?’. Possible responses were: ‘No’, ‘Yes, Quite choosy’, or ‘Yes, very choosy’. This single question is similar to those used in several recent studies (Goh & Jacob, 2012; Jani Mehta, Mallan, Mihrshahi, Mandalika, & Daniels, 2014; Orun, Erdil, Cetinkaya, Tufan, & Yalcin, 2012).

Longitudinal classification was used to identify groups with picky eating behaviour for further analysis. Children aged 2–5.5 years with *early onset PPE* comprised those for whom ‘Yes, very choosy’ was recorded for at least three out of the four time points. The *early onset Non-PPE* group included children for whom ‘Yes, very choosy’ was selected for two out of the four timepoints, with the first occasion being reported at 2 or 3 years old. The children for whom ‘No’ was selected for all four timepoints were characterised as *never picky eaters* (NPE). Children who were *occasional picky eaters* (n = 4530) were excluded from this analysis. Further information on the classification of picky eating behaviour can be found elsewhere (Taylor, Hays, & Emmett, 2019).

### 2.4 Study size

**Fig. 1:**
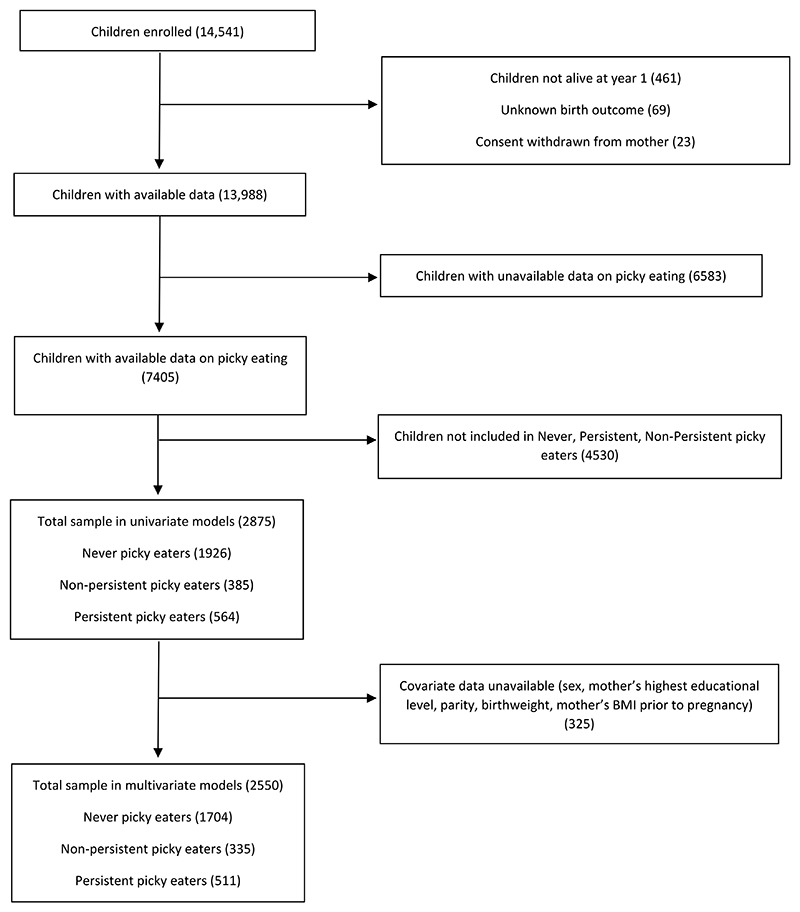
Flow chart of study sample.

In total, 14,541 pregnant women were enrolled into ALSPAC, and 14,062 births were recorded, from which 13,988 children were alive at 1 year of age (Boyd et al., 2013). The sociodemographic characteristics of this cohort were similar at baseline with the corresponding UK census data of that time (Fraser et al., 2013) After excluding the children with unknown birth outcome, maternal consent withdrawn, unavailable data on picky eating over time and lacking later feeding behaviour information or with a classification of occasional picky eating, a total of 2875 participants were included in this analysis (2550 in the adjusted models) (see study flowchart Fig. 1).

### 2.5 Feeding difficulties

The primary caregiver completed postal questionnaires including questions on their child’s feeding behaviour at 5.5, 6.5, 7 and 8.5 years old. Questions at 5.5 years included: the child’s feeding behaviours, involvement in food preparation practices, behaviour around meal-times, and the child’s or parent’s perceptions around meals. Questions assessed longitudinally (asked at each time point) included feeding difficulties, problematic feeding behaviours, and practices that the mother used to deal with the child’s feeding difficulties. Details of the questions and answer categories are given in the Supplementary text.

### 2.6 Maternal early worry and the difficulty with feeding the child

The mother was asked the following question 15 months after the child’s birth through a postal questionnaire: ‘Since your child was 6 months old has he/she at any time been choosy with food?’ and if the answer was ‘Yes’ the mother indicated if she was ‘Worried’ or ‘Not worried’. Questions about difficulties with feeding the child were also asked at age 5.5, 7 and 8.5 years old. Further details are given in the Supplementary text.

### 2.7 Confounders

Questionnaires in pregnancy and after the birth of the child were used to obtain maternal and child-based variables. Variables that were associated with the exposure and not on the causal pathway were included as confounders in all the adjusted models along with sex, which has shown inconsistent associations with picky eating previously (Taylor & Emmett, 2019). These variables included the mother’s pre-pregnancy body mass index (continuous), parity (0, ≥1), age at delivery (≤24, 25–29, ≥30 years), length of pregnancy (continuous, weeks) and the child’s ethnic background (categorised into white or non-white; response categories were White/Black, Caribbean/Black, African/-Other, Black/Indian Pakistani/Bangladeshi/Chinese/Other; the baseline ALSPAC cohort contained 94.1% white with the other categories all <1%). The mother’s highest educational attainment (categorised into: None/Vocational/Ordinary Level, Certificate of School Education usually taken at 16 years of age/Advanced Level Certificate usually taken at 18 years of age/Degree).

### 2.8 Statistical analysis

Statistical analysis was carried out with STATA version 16.0. All available cases were included in the analysis to maximise power.

ANOVA was used for comparisons of the baseline characteristics of the three picky eating groups, as well as the comparison between the sample that were included and those that were excluded. Statistical comparisons were made using multiple comparisons (Bonferroni correction).

Multinominal logistic regression models (outcome with more than two values) with the picky eating status of a child at preschool (PPE vs NPE; Non-PPE vs NPE) as the independent variable, and the feeding behaviour as the dependent variable, were created. Binary logistic regression models (dichotomous outcome) were used to evaluate the likelihood of feeding difficulties occurring at 5.5 years (reference category in the outcome was the category with that included the most participants) and 7 or 8.5 years, depending on picky eating group. Binary logistic regression models were used to compare maternal feeding strategies and later difficulty in feeding. All the regression models were adjusted for sex of the child and the confounders that differed between the picky eating groups in univariate analysis. Likelihood ratio tests were used to assess the goodness of fit of adjusted models based on the critical value from the degrees of freedom.

The effect of early maternal worry on moderating the association between persistent picky eating (PPE vs NPE or Non-PPE vs NPE) and later feeding difficulties was evaluated in binary logistic regression models: an interaction term between the early maternal worry and being a picky eater (PPE vs NPE or Non-PPE vs NPE) was included in the adjusted models. A moderating effect was accepted if: (1) both the exposure and moderator were associated with feeding difficulty in the adjusted model; and (2) the interaction term was significant in the adjusted model.

## 3 Results

Children included in the study differed from those excluded in having higher maternal educational attainment, older delivery age and longer duration of pregnancy. In addition, the children’s ethnic background was more likely to be white and the mean birthweight was greater (Supplementary Table 1).

Of the 7405 children with data on picky eating, 4530 were classified as occasional picky eaters and were not included in these analyses. Of the children with data, 1926 were NPE (26%), 385 Non-PPE (5%) and 564 PPE (8%). The demographic characteristics of these three groups are compared in Table 1. There were no differences between NPE and Non-PPE, or between PPE and Non-PPE. Children classified as PPE had lower mean birthweight and were more often first born, and their mothers had higher educational attainment and lower pre-pregnancy BMI than mothers of children who were NPE. Non-PPE children had lower mean birthweight and their mothers had lower pre-pregnancy BMI than mothers of children who were NPE.

Adjusted associations of food-related behaviours at age 5.5 and picky eating category are presented in Table 2 (unadjusted analyses are shown in Supplementary Table 2). PPE and Non-PPE were 235 and 37 times as likely not to enjoy eating and 61 and 27 times as likely to not finish all food on their plates, respectively, than NPE. PPE were 96 and Non-PPE 25 times as likely to never try different foods than NPE. They were also 49 and 17 times as likely to play with food most times, respectively, than NPE.

The analysis of the parental-directed behaviours showed that children who were PPE or Non-PPE were slightly more likely to assist their mother with food selection from the cupboard/fridge than NPE. Children who were PPE or Non-PPE were more likely not to help their mother prepare the table/meal for eating or not to help with cooking/food preparation than NPE. The family were also less likely to have a proper cooked meal every day if a picky eater was present.

During mealtimes, children who were PPE or Non-PPE had higher odds of participating in mealtimes considered to be a rush, or with children arguing or adult-child arguments than NPE. PPE and Non-PPE and their parents were 18 and 4 times as likely to participate in meal-times that were not enjoyable for everyone, respectively, than NPE.

Feeding difficulties associated with the child’s picky eating classification are presented in Table 3 (unadjusted and minimally adjusted analyses are shown in Supplementary Tables 3 and 4). Both PPE and Non-PPE were more likely to have most of the problematic behaviours at age 5.5, 7 and 8.5 years (being difficult to feed, deliberately not eat enough, refuse to eat, being choosy, and not getting into an eating routine easily) compared with NPE, with the likelihood showing a decreasing trend as the children grew older. Most notably, PPE had very high odds of being choosy and difficult to feed at the age of 5.5 but these odds had decreased considerably 3 years later. At each age children who were Non-PPE had lower odds of these behaviours than PPE. Overeating was inversely associated with a child being PPE or Non-PPE compared with NPE at each age.

In the investigation of the moderating effect of early maternal worry on feeding behaviours, ‘difficulties in eating what the mother wanted’ was consistently associated with maternal worry and both PPE and Non-PPE and at 5.5, 7 and 8.5 years old (Supplementary Table 5). The interaction term PPE × maternal worry was only significant at 8.5 years. The interaction term Non-PPE × maternal worry was significant at both 7 and 8.5 years old. Other variables for feeding behaviours were not associated with both PPE or Non-PPE and with maternal worry at any age. There was no evidence of a consistent moderation of feeding behaviours by early maternal worry.

The impact of maternal strategies for feeding her child at 5.5 years of age in PPE and Non-PPE combined on feeding difficulties at age 6.5 and 8.5 years are presented in Table 4 (unadjusted analyses are shown in Supplementary Table 6). Mothers who let the child eat something else (only at 8.5 years), or encouraged the child to eat using rewards or with a game/story, were more likely to face difficulties in feeding the child up to 3 years later than those who did not use these techniques at 5.5 years. There was no evidence that any of the strategies investigated alleviated the mother’s difficulty in feeding the child.

## 4 Discussion

About one in seven children were classified as long-term picky eaters during preschool, with more than half of these being classified as PPE. These children tended to be more food neophobic, play with or leave food on their plates, and enjoy eating less at 5.5 years of age than children who were NPE. They tended to be less involved in preparing food or clearing after meals and had less enjoyable mealtimes which often included arguments with other children or their parents. Children who were PPE or Non-PPE tended to be more challenging to feed, deliberately not eat enough, refuse to eat, be choosy and be difficult to get into an eating routine, but were less likely to overeat through their early school years compared with those who were NPE. These behaviours were more prominent at the beginning of school (5.5 years of age) and tended to decrease gradually over the next 3 years. Most of the feeding behaviours and difficulties were substantially more common in PPE than Non-PPE from 5.5 to 8.5 years of age. There was no consistent evidence of a moderating effect of early maternal worry on later feeding difficulties in PPE or Non-PPE. Maternal strategies at 5.5 years, such as encouragement to eat with stories/games or rewards or allowing the child to eat something else were associated with increased difficulties in feeding up until 8.5 years of age. None of the practices investigated alleviated the problem, including mixing with other food or adding sauce, not making it an issue or trying the food on a different day.

This study has confirmed our initial hypothesis that children who are PPE will display more troublesome feeding behaviours in early school years than those who are NPE or children who resolved their pickiness early (Non-PPE). Contradicting our hypothesis, children whose pickiness seems to have resolved early can still display troublesome feeding behaviours in early school years although to a lesser extent than those with unresolved pickiness.

This continued difficulty in feeding in some picky eaters can be a source of parental concern and child discomfort, and has been shown to be a mutual psychological burden in some studies (Emmett et al., 2018; Ong, Phuah, Salazar, & How, 2014; Wolstenholme, Kelly, Hennessy, & Heary, 2020). The results of our study suggests that parents can be offered reassurance that these problematic feeding behaviours are likely to resolve over time. Our previous work in this cohort of children has shown that growth up to 17 years of age in children with PPE is within the normal range (Taylor, Steer, Hays, & Emmett, 2019) and that differences in food and nutrient intakes between PPE and NPE also tend to resolve (Taylor, Hays, & Emmett, 2019). By 13 years of age the only dietary differences were that PPE children have lower intakes of vegetables and higher intakes of free sugars than NPE.

Children who were PPE in this study were likely to be neophobic and less happy during meals: this is not surprising since these behaviours often characterise picky eating (Dovey et al., 2008; Taylor & Emmett, 2019). Children who are picky eaters have been shown to eat less than those who are not (Taylor, Hays, & Emmett, 2019; Tharner et al., 2014; Volger et al., 2017), and as expected, PPE tended to avoid eating by not finishing the food on their plate and playing with their food. Mealtimes with picky eaters were stressful often including arguments, again confirming previous studies (Emmett et al., 2018; Hafstad et al., 2013; Ong et al., 2014; Wolstenholme et al., 2020). A previous longitudinal study has also found that the struggle in feeding the child is greater among PPE than Non-PPE (Mascola et al., 2010). PPE and Non-PPE showed a similar avoidance of cooking or setting the table although they were equally likely to assist in selecting which foods to eat. It may be that involving them in cooking would be helpful in reducing picky eating behaviour (Levene & Williams, 2018).

Choosiness and difficulty in feeding in the children were identified by parental questionnaires during pre-school age. Although both choosiness and difficulty in feeding tended to decrease over time, they remained a major concern. These results confirm previous findings regarding the continuation of feeding difficulties and choosiness in middle to late childhood in some children with early picky eating behaviour (Kauer et al., 2015; Mascola et al., 2010). Our findings on behaviour, such as deliberately not eating enough and refusing what is offered were similar to those in an earlier study (Kotler et al., 2001; Marchi & Cohen, 1990). Overeating was less likely among PPE. Rarely, the combination of not overeating, refusing to eat what is offered or deliberately not eating enough over time together with parental concern may increase the likelihood of a child developing an eating disorder (Kotler et al., 2001; Marchi & Cohen, 1990), suggesting a possible association between picky eating and symptoms of anorexia nervosa in adolescence (Marchi & Cohen, 1990). Childhood picky eating may also develop into adult picky eating, which is associated with symptoms of depression, social eating anxiety, psychological inflexibility, lower earning-related quality of life and pervasive developmental problems (Cardona Cano et al., 2016; Ellis, Galloway, Webb, & Martz, 2017; Kauer et al., 2015). The complex relations between child characteristics, parent feeding beliefs, feeding practices and awareness, and the emotional climate at mealtimes have been described based on an ethnographic analysis of qualitative studies (Wolstenholme et al., 2020). Our study has confirmed that children who are PPE continue to present difficult feeding behaviours at school age such that their parents are likely to need ongoing support in feeding their child.

Parental worry is common among parents of children with picky eating behaviour (Dovey et al., 2008) and can be accompanied by feeding difficulties for many years (Mascola et al., 2010; Wolstenholme et al., 2020). In our previous analysis, early maternal worry about their child’s choosiness was shown to predict the child’s picky eating behaviour at 3 years (Emmett et al., 2018). However, we found no substantial evidence that early maternal worry moderated the effect on feeding behaviours at school age of a child having picky eating behaviour. It is possible that worried mothers use pressure to eat when feeding their child and this has been associated with an increase in choosiness and feeding difficulties (Cardona Cano et al., 2015; Dovey et al., 2008; Levene & Williams, 2018). If the child is picky throughout preschool years, the mother will probably have become accustomed to using certain strategies, so is not likely to change her habits during school years.

We found that practices such as allowing the child to eat another food and offering rewards during feeding difficulties were associated with increased future difficulties in feeding. These practices have been shown to be harmful and should be avoided (Levene & Williams, 2018; Taylor & Emmett, 2019). Encouraging the child with a story/game, unexpectedly, increased the likelihood of feeding difficulties in school age. Making the meal a fun and interactive activity is often advised (Levene & Williams, 2018). Furthermore, practices that are commonly advised by healthcare providers such as avoiding judgment or pressure to eat, or offering the same food again (Levene & Williams, 2018) did not seem to be protective in this study. It is likely that by 5.5 years most parents will already have tried and decided to discontinue practises with no short-term results. It is also possible that these children, who were picky from a young age, have very set habits that are difficult to change whatever tactics the parents use (Jansen et al., 2017).

The main strength of this study is that we were able to include a large cohort of children and their parents, with an extensive range of data on feeding behaviours and practices related to picky eating. These data allowed us to classify the children by the persistence of their picky eating behaviour. Several studies have evaluated feeding behaviours associated with picky eating, but often with limited sample numbers (Cardona Cano et al., 2015; Jansen et al., 2017; Orun et al., 2012), and only a few have distinguished picky eating by persistence of picky eating (Mascola et al., 2010; Toyama & Agras, 2016). Our findings extend knowledge of the aetiology and consequences of picky eating in school-age children.

The picky eating behaviour of the children was assessed longitudinally over four age points and was defined using a non-direct question, allowing the parents’ answers to determine their child’s pickiness. In contrast, some recent studies have used a range of questions contained in standardised questionnaires. The method of identification of picky eating status used here is relatively simple compared with alternative methods (Toyama & Agras, 2016), but similar classifications in terms of simplicity have been used in other studies (Goh & Jacob, 2012; Jani, Mallan, & Daniels, 2015; Mascola et al., 2010). The longitudinal PE classification was similar to that used by Cardona Cano et al. (2015). We were able to make comparisons with children that had never displayed picky behaviour through preschool, and this is likely to have increased our chances of finding large differences with the picky eating groups. We were also able to assess a large range of potential confounders and include those that differed between picky eating groups in all analyses.

Our study also has limitations. The picky eating status of the child was based on parental perception and not a professional’s judgement, and the question asked did not cover the full range of picky eating traits that are defined in other studies. Despite this, the longitudinal nature of the classification is valuable as it is a unique feature of the ALSPAC study. The questionnaires were completed by the parents without the assistance of researchers. The generalisability of findings may be limited as only children living in a prescribed geographical area were included, with a limited representation of participants with ethnic minority background. Parental practices related to feeding difficulties were asked about when the children were 5.5 years of age and not as soon as picky eating appeared. There may also have been residual confounding that we were unable to account for. A further limitation is the amount of missing data. Analysis of differences between participants with or without available data has shown some differences in maternal characteristics (Rogelberg & Luong, 1998). We also excluded the children who were occasional picky eaters because we considered it important to have a clear distinction between children who were never picky and those who were often picky (Taylor, Hays, & Emmett, 2019). Over-adjustment can lead to biased estimates with loss of precision, but we were careful to include only those variables that were on the causal pathway and were not mediators (Schisterman, Cole, & Platt, 2009). Finally, we found some very high odds ratios with wide confidence intervals possibly due to the small sample size in some of the groups: this indicates less precise estimates.

## 5 Conclusion

We have highlighted the importance of early identification of children with persistent picky eating behaviour, as feeding difficulties can remain through school age and potentially affect long-term growth, development, and the child’s and parents’ mental health. We found that even children who seemed to resolve their pickiness before starting school can have difficulties related to feeding during school years. However, the likelihood of the difficulties declined with age. Parents should be reassured that if their child exhibits picky eating behaviours as they start school (1 in 7 did so in this study) it is very likely that these behaviours will slowly resolve over time. In previous studies, at 13 years of age there were still differences in diet in PPE compared with NPE children (lower vegetable and higher free sugars intakes), but growth in children who were picky eater up to age 17 years was within the normal range in the ALSPAC cohort. Overall, our studies show that being a PPE during early childhood is not likely to lead to permanent problems. If parents are worried about their child’s growth or their child insists on a very restricted diet, they should seek professional advice.

## Supplementary Material

Supplementary Materials

## Figures and Tables

**Table 1:** Maternal and child baseline characteristics for children who were NPE, PPE or Non-PPE during the first 5 years of life.

Characteristics	Category	NPE	PPE	Non-PPE	p value			
					NPE vs PPE	NPE vs Non-PPE	PPE vs Non-PPE	
Participant sex	Male	980 (50.9%)	304 (53.9%)	207 (53.8%)	0.622	0.904	1.000	
	Female	946 (49.1%)	260 (46.1%)	178 (46.2%)				
Mother’s highest educational attainment	CSE/Vocational/O level	1159 (61.2%	301 (54.0%)	232 (61.9%)	0.007	1.000	0.050	
	A level/Degree	734 (38.8%)	256 (46.0%)	143 (38.1%)				
Child ethnic background	White	1811 (97.2%)	528 (97.2%)	358 (96.0%)	1.000	0.668	0.815	
	Non-white	53 (2.8%)	15 (2.8%)	15 (4.0%)				
Mother’s age at delivery (years)	16–24	314 (16.3%)	82 (12.5%)	53 (13.8%)	0.206	1.000	1.000	
	25–29	790 (41.0%)	216 (33.0%)	165 (42.9%)				
	30–43	822 (42.7%)	266 (40.7%)	167 (43.4%)				
Parity	≥1	1068 (57.0%)	263 (47.7%)	187 (49.6%)	<0.001	0.025	1.000	
	0	805 (43.0%)	288 (52.3%)	190 (50.4%)				
Birthweight (g)	n	1909	554	382	0.001	0.023	1.000	
	Mean ± SD	3473 ± 526	3384 ± 521	3394 ± 535				
Length of pregnancy (weeks)	n	1926	564	385	1.000	0.757	1.000	
	Mean ± SD	39.5 ± 1.8	39.5 ± 1.6	39.6 ± 1.7				
Pre-pregnancy BMI (kg/m^2^)	n	1768	531	348	0.008	0.001	1.000	
	Mean ± SD	23.2 ± 3.8	22.6 ± 3.6	22.4 ± 3.2				

NPE, never picky eater; PPE, persistent picky eater; Non-PPE, non-persistent picky eater.

For categorical analyses the number of participants (%) and for continuous the mean ± SD is shown.

Significant differences between the three groups were calculated using ANOVA with multiple comparisons correction (Bonferroni).

**Table 2:** Comparison of food-related behaviours, parent-directed behaviours and mealtimes at 5.5 years between children who were PPE and NPE, and between children who were Non-PPE and NPE (adjusted analyses).

	Behaviour/perception	Answer	NPE vs PPE				NPE vs Non-PPE			
			n	OR (95% CI)	p value		n	OR (95% CI)	p value	
**Food-related** ** behaviours**	Child likes to try different foods	Yes, most of the time	835	0.13 (0.07, 0.25)	<0.001		846	0.17 (0.11, 0.26)	<0.001	* *
		Yes, sometimes	913	Ref.			964	Ref.		
		No, not at all	454	95.8 (64.7, 141.8)	<0.001		219	24.8 (16.9, 36.3)	<0.001	* *
	Child seems to enjoy eating	Yes, most of the time	1618	Ref.			1636	Ref.		
		Yes, sometimes	478	18.7 (14.3, 24.4)	<0.001		372	10.0 (7.62, 13.1)	<0.001	* *
		No, not at all	109	235.4 (100.6, 551.1)	<0.001		25	36.9 (14.3, 95.6)	<0.001	* *
	Child plays with food rather than eating eagerly	Yes, most of the time	135	48.5 (29.8, 79.0)	<0.001		61	16.5 (9.33, 28.4)	<0.001	* *
		Yes, sometimes	645	7.18 (5.64, 9.14)	<0.001		547	4.65 (3.59, 6.03)	<0.001	* *
		No, not at all	1421	Ref.			1416	Ref.		
	Child finishes all the food on plate	Yes, most of the time	1267	Ref.			1263	Ref.		
		Yes, sometimes	782	7.59 (5.86, 9.84)	<0.001		677	4.98 (3.78, 6.56)	<0.001	* *
		Not at all	158	61.1 (38.6, 96.7)	<0.001		90	26.91 (16.34, 44.32)	<0.001	* *
**Parental directed** ** behaviours**	Child helps to choose food from cupboard/ fridge	Yes, often	572	1.40 (1.11, 1.76)	0.004		514	1.34 (1.02, 1.77)	0.035	
		Yes, sometimes	1263	Ref.			1169	Ref.		
		Never/rarely	368	1.00 (0.76, 1.33)	0.976		347	1.13 (0.82, 1.57)	0.449	
	Child comes shopping with mother and helps choose food	Yes, often	566	1.25 (0.98, 1.57)	0.067		496	0.89 (0.66, 1.19)	0.421	
		Yes, sometimes	1222	Ref.			1148	Ref.		
		Never/rarely	418	1.02 (0.78, 1.33)	0.891		387	0.90 (0.65, 1.23)	0.493	
	Child helps with cooking/food preparation	Yes, often	202	0.71 (0.47, 1.08)	0.107		200	1.15 (0.75, 1.77)	0.517	
		Yes, sometimes	1320	Ref.			1215	Ref.		
		Never/rarely	683	1.86 (1.50, 2.30)	<0.001		616	2.09 (1.62, 2.70)	<0.001	* *
	Child helps get things for meal/sets table	Yes, often	722	0.65 (0.51, 0.82)	<0.001		701	0.89 (0.68, 1.16)	0.399	
		Yes, sometimes	1308	Ref.			1186	Ref.		
		Never/rarely	173	2.63 (1.89, 3.65)	<0.001		142	2.61 (1.77, 3.83)	<0.001	* *
	Child helps clear up after meals	Yes, often	506	0.66 (0.50, 0.87)	0.003		496	0.96 (0.71, 1.31)	0.815	
		Yes, sometimes	1206	Ref.			1100	Ref.		
		Never/rarely	489	1.60 (1.27, 2.03)	<0.001		430	1.66 (1.26, 2.20)	<0.001	* *
	Family has proper cooked meal every day	Yes	1890	Ref.			1769	Ref.		
		No	301	2.15 (1.65, 2.79)	<0.001		248	1.55 (1.12, 2.16)	0.009	
**Mealtimes**	Mealtimes are enjoyable for everyone	Mostly/quite often	1650	Ref.			1611	Ref.		
		Occasionally	511	5.00 (3.99, 6.25)	<0.001		393	3.03 (2.32, 3.94)	<0.001	* *
		Never	32	23.2 (9.79, 54.8)	<0.001		12	5.11 (1.59, 16.5)	0.006	
	Mealtimes are a rush	Mostly/quite often	190	1.81 (1.30, 2.52)	<0.001		169	1.77 (1.21, 2.59)	0.004	
		Occasionally	1409	Ref.			1310	Ref.		
		Never	581	1.21 (0.96, 1.53)	0.103		522	1.02 (0.77, 1.36)	0.875	
	Mealtimes give time to talk to each other	Mostly/quite often	1584	Ref.			1507	Ref.		
		Occasionally	535	2.37 (1.90, 2.96)	<0.001		459	1.88 (1.44, 2.45)	<0.001	* *
		Never	72	4.64 (2.85, 7.55)	<0.001		51	2.47 (1.32, 4.64)	0.005	
	Mealtimes include arguments between the children	Mostly/quite often	314	2.08 (1.58, 2.73)	<0.001		270	1.82 (1.31, 2.52)	<0.001	* *
		Occasionally	1164	Ref.			1087	Ref.		
		Never	694	0.91 (0.71, 1.16)	0.442		644	0.89 (0.67, 1.18)	0.409	
	Mealtimes include arguments between adults & children	Mostly/quite often	145	2.19 (1.53, 3.15)	<0.001		120	1.96 (1.28, 3.00)	0.002	
		Occasionally	1081	Ref.			980	Ref.		
		Never	955	0.60 (0.48, 0.74)	<0.001		904	0.64 (0.49, 0.82)	0.001	

NPE, never picky eater; PPE, persistent picky eater; Non-PPE, non-persistent picky eater.

Adjusted multinomial logistic regression.

The picky eating reference category is always NPE in both comparisons. The exposure reference category is always the most frequent answer, shown as ‘Ref.’. All analyses were adjusted for maternal educational attainment, pre-pregnancy BMI, and child’s sex, parity and birthweight.

**Table 3:** Feeding behaviours at school age in children who were PPE or Non-PPE compared with those who were NPE during the first 5 years of life (adjusted analyses).

Age	Questions: During the past year the child …	Response	PPE vs NPE				Non-PPE vs NPE			
			n	OR (95% CI)	p value		n	OR (95% CI)	p value	
**5.5 years**	Indicated difficulties in eating what the mother wanted	No	1293				1327			
		Yes	915	80.4 (49.6, 130.5)	<0.001		710	16.4 (11.9, 22.5)	<0.001	
	Has deliberately not eaten sufficient amount of food	No	1897				1805			
		Yes	302	5.85 (4.50, 7.59)	<0.001		226	4.19 (3.09, 5.67)	<0.001	
	Has refused to eat the offered food	No	1200				1236			
		Yes	1003	44.2 (29.1, 67.0)	<0.001		793	10.4 (7.7, 14.1)	<0.001	
	Has been choosy with food	No	955				972			
		Yes	1252	126.1 (52.0, 306.1)	<0.001		1060	17.6 (11.3, 27.5)	<0.001	
	Has over-eaten	No	1820				1636			
		Yes	387	0.21 (0.14, 0.32)	<0.001		395	0.44 (0.30, 0.63)	<0.001	
	Was difficult to get into eating routine	No	1953				1885			
		Yes	242	23.3 (16.5, 33.0)	<0.001		136	13.6 (9.21, 20.0)	<0.001	
**7 years**	Indicated difficulties in eating what the mother wanted	No	1199				1235			
		Yes	813	37.2 (26.0, 53.1)	<0.001		607	10.2 (7.63, 13.6)	<0.001	
	Has deliberately not eaten sufficient amount of food	No	1725				1624			
		Yes	309	4.44 (3.44, 5.74)	<0.001		238	3.50 (2.58, 4.74)	<0.001	
	Has refused to eat the offered food	No	1115				1136			
		Yes	919	15.5 (11.5, 20.8)	<0.001		726	6.42 (4.86, 8.48)	<0.001	
	Has been choosy with food	No	925				938			
		Yes	1109	52.1 (29.7, 91.3)	<0.001		924	15.9 (10.5, 24.1)	<0.001	
	Has over-eaten	No	1652				1497			
		Yes	382	0.41 (0.30, 0.57)	<0.001		365	0.41 (0.28, 0.61)	<0.001	
	Was difficult to get into eating routine	No	1831				1759			
		Yes	203	15.0 (10.6, 21.3)	<0.001		103	6.71 (4.42, 10.2)	<0.001	
**8.5 years**	Indicated difficulties in eating what the mother wanted	No	1173				1209			
		Yes	664	22.6 (16.7, 30.5)	<0.001		481	7.57 (5.72, 10.0)	<0.001	
	Has deliberately not eaten sufficient amount of food	No	1630				1525			
		Yes	193	4.50 (3.29, 6.16)	<0.001		150	3.62 (2.52, 5.21)	<0.001	
	Has refused to eat the offered food	No	1139				1157			
		Yes	684	14.1 (10.7, 18.6)	<0.001		519	6.41 (4.85, 8.47)	<0.001	
	Has been choosy with food	No	862				884			
		Yes	968	30.4 (19.3, 47.9)	<0.001		797	8.76 (6.21, 12.4)	<0.001	
	Has over-eaten	No	1480				1339			
		Yes	341	0.50 (0.36, 0.70)	<0.001		333	0.68 (0.48, 0.98)	0.037	
	Was difficult to get into eating routine	No	1684				1607			
		Yes	133	14.5 (9.42, 22.2)	<0.001		66	6.89 (4.12, 11.5)	<0.001	

NPE, never picky eater; PPE, persistent picky eater; Non-PPE, non-persistent picky eater.

The picky eating reference category is always NPE in both comparisons. The empty cells in the OR (95% CI) and p value represent the reference category (‘No’).

All analyses adjusted for maternal educational attainment, pre-pregnancy BMI, and child’s sex, parity and birthweight.

**Table 4:** Effect of maternal strategies for feeding her child at age 5.5 years on the presence of feeding difficulties at age 6.5 and 8.5 years in children who were PPE or Non-PPE combined (adjusted analyses).

Maternal strategies to improve feeding behaviour when she was faced with feeding difficulties in the child aged 5.5 years		Is the child difficult to feed?							
		Age 6.5 years				Age 8.5 years			
		n	OR (95% CI)	p value		n	OR (95% CI)	p value	
Mother lets the child eat something else	No	216	Ref			201	Ref		
	Yes	554	1.5 (0.97, 2.34)	0.069		505	1.8 (1.23, 2.63)	0.002	
Mother encourages child to eat the food by making up a game or story	No	401	Ref			371	Ref		
	Yes	370	2.42 (1.54, 3.79)	<0.001		336	1.62 (1.12, 2.34)	0.010	
Mother mixes food with other food that the child likes and will eat	No	497	Ref			459	Ref		
	Yes	277	1.14 (0.74, 1.77)	0.555		251	1.15 (0.78, 1.68)	0.482	
Mother lets child put sauce on food to cover up the taste/appearance	No	383	Ref			345	Ref		
	Yes	388	1.39 (0.91, 2.11)	0.125		362	1.31 (0.91, 1.88)	0.140	
Mother tries to persuade the child to eat a very small amount	No	30	Ref			28	Ref		
	Yes	746	2.05 (0.85, 4.93)	0.110		683	2.07 (0.93, 4.61)	0.075	
Mother does not let child to leave table/have anything else until it finishes its plate	No	530	Ref			490	Ref		
	Yes	242	1.16 (0.74, 1.84)	0.514		218	0.97 (0.66, 1.43)	0.887	
Mother tries to encourage child with rewards if it finishes the food	No	389	Ref			363	Ref		
	Yes	387	1.78 (1.16, 2.74)	0.008		348	1.53 (1.06, 2.21)	0.022	
Mother takes the food away and gives something else to eat	No	346	Ref			318	Ref		
	Yes	428	1.23 (0.81, 1.86)	0.337		390	1.15 (0.80, 1.65)	0.441	
Mother does not make issue of child not eating the food	No	44	Ref			37	Ref		
	Yes	730	1.99 (0.95, 4.17)	0.069		671	0.69 (0.28, 1.7)	0.421	
Mother tries same food again on a different day	No	140	Ref			131	Ref		
	Yes	633	1.24 (0.74, 2.08)	0.407		578	1.51 (0.98, 2.34)	0.062	

NPE, never picky eater; PPE, persistent picky eater; Non-PPE, non-persistent picky eater.

Adjusted binary logistic regression. Values are presented as odds ratio (95% CI).

All analyses are adjusted for maternal educational attainment, pre-pregnancy BMI, and child’s sex, parity and birthweight. Three children who were PPE and three who were non-PPE had mothers who replied with “Never difficult” when asked about the strategies they used and were excluded from the analysis.

## Data Availability

Data will be made available on request.

## Data sharing statement

Data will be shared with bona fide researchers on application to the ALSPAC Executive Committee (https://www.bristol.ac.uk/alspac/researchers/access/).

## Abbreviations

Non-PPE: non-persistent picky eater
NPE: never picky eater
PPE: persistent picky eater
