# Supplementary material for: Effect of being a persistent picky eater on feeding difficulties in school-aged children
Source: Appetite. Author manuscript; Available in PMC 2024 Jul 28. (PMC7616308; doi:10.1016/j.appet.2023.106483)
Supplement: Supplementary Materials [file EMS197622-supplement-Supplementary_Materials.docx]

**Supplementary text: outcome variables**

| **Questions** | **Response categories** | **Derived variables** |
| --- | --- | --- |
| **Food-related behaviours (5.5 years)** |  |  |
| How far to the following statements describe your child?   - She likes to try different foods - She seems to enjoy eating - She plays with her food rather than eating eagerly - She finishes all the food on her plate | Yes, most of the time  Yes, sometimes  No, not at all | - |
| **Parental-directed behaviours (5.5 years)** |  |  |
| - She helps choose what we have by looking in cupboard/fridge - She comes shopping with me and helps choose the food we buy - She helps with the cooking/preparation - She helps to get things out for the meal/sets the table - She helps to clear things away after the meal | Yes, most of the time  Yes, sometimes  No, not at all | - |
| **Mealtimes (5.5 years)** |  |  |
| - Mealtimes are enjoyable for everyone - Mealtimes are a rush - Mealtimes give us time to talk to each other - Mealtimes include arguments between adults and children - Mealtimes include arguments between adults | Never  Occasionally  Quite often  Mostly | Never  Occasionally  Mostly/Quite often |
| **Child’s feeding behaviours (5.5, 7 and 8.5 years)** |  |  |
| In the past year have you had any difficulties getting her to eat what you wanted her to? | Yes, great difficulty  Yes, some difficulty  Yes, occasional difficulty  No, no difficulty | Yes = Yes, great difficulty, Yes, some difficulty, Yes, occasional difficulty  No = No, no difficulty |
| In the past year has she at any time:   - Deliberately not eaten enough food - Refused to eat the food you think she should - Been choosy with food - Over-eaten - Been difficult to get into an eating routine | Yes, worried me greatly  Yes, worried me a bit  Yes, did not worry me  No, did not happen | Yes = Yes, worried me greatly/Yes, worried me a bit  No = Yes, did not worry me |
| **Early maternal worry (15 months)** |  |  |
| - Is your child choosy with food? | Yes, worried me greatly  Yes, worried me a bit  Yes, did not worry me  No, did not happen | Yes = Yes, worried me greatly/  Yes, worried me a bit  No = Yes, did not worry me |
| **Parental strategies (6.5 and 8.5 years)** |  |  |
| Children are often difficult about eating certain foods. When your study child is difficult about a certain food you would like her to eat, how far do the following sentences describe how you deal with this?   - I let her choose something else - I try to encourage her to eat the food by making up a game or story - I mix the food with another food that she likes and will eat - I let her put sauce on the food to cover up the taste/appearance - I try to persuade her to eat just a very small amount of the food - I do not let her leave the table or have anything else to eat until she eats all the food on the plate - I try to encourage her by offering a reward (e.g. a sweet or new toy) if she finishes the food - If she will not eat a certain food I take it away and give her something else to eat - I try not to make an issue of it - I try the same food on a different day | Yes, most of the time  Yes, sometimes  No, not at all  Never difficult | Yes = Yes, most of the time/Yes, sometime  No = No, not at all |

ALSPAC questionnaires are available online: <http://www.bristol.ac.uk/alspac/researchers/our-data/questionnaires/>

**Supplementary Tables**

**Supplementary Table 1**Maternal and child baseline characteristics of: (1) included sample (Never, Persistent and Non-Persistent Picky Eaters) in comparison with the excluded sample (no data on picky eating or occasional picky eaters only); and (2) included sample in comparison with the occasional picky eaters

| **Characteristics** | **Category** | **Excluded** | **Included (NPE, PPE, Non-PPE)** | **Occasional picky eaters** | **P value** | |
| --- | --- | --- | --- | --- | --- | --- |
|  |  |  |  |  | **Excluded vs Included (NPE, PPE, Non-PPE)** | **Included (NPE, PPE, Non-PPE) vs Occasional picky eaters** |
| Participant sex | Male | 5714 (51.6%) | 1491 (51.9%) | 2287 (50.5%) | 0.778 | 0.249 |
|  | Female | 5367 (48.4%) | 1384 (48.1%) | 2243 (49.5%) |  |  |
| Mother’s highest educational attainment | CSE/Vocational/O level | 6324 (66.1%) | 1692 (59.9%) | 2519 (56.6%) | <0.001 | 0.005 |
|  | A level/Degree | 3248 (33.9%) | 1133 (40.1%) | 1934 (43.4%) |  |  |
| Child ethnic background | White | 8757 (94.3%) | 2697 (97.0%) | 4217 (96.2%) | <0.001 | 0.058 |
|  | Non-white | 526 (5.7%) | 83 (3.0%) | 168 (3.8%) |  |  |
| Mother’s delivery age group (years) | 16-24 | 2879 (26.0%) | 449 (15.6%) | 643 (14.2%) | <0.001 | 0.018 |
|  | 25-29 | 4227 (38.2%) | 1171 (40.7%) | 1789 (39.5%) |  |  |
|  | 30-43 | 3952 (35.7%) | 1255 (43.7%) | 2093 (46.3%) |  |  |
| Parity | ≥1 | 5625 (55.7%) | 1518 (54.2%) | 2369 (53.5%) | 0.166 | 0.537 |
|  | 0 | 4480 (44.3%) | 1283 (45.8%) | 2063 (46.5%) |  |  |
| Birthweight (g) | n | 10931 | 2845 | 4472 | <0.001 | 0.815 |
|  | Mean±SD | 3377.9±566.5 | 3445.1±527.3 | 3448.1±525.4 |  |  |
| Length of pregnancy (weeks) | n | 11081 | 2875 | 4530 | <0.001 | 0.855 |
|  | Mean±SD | 39.4 ±2.0 | 39.5±1.8 | 39.5±1.8 |  |  |
| Pre-pregnancy BMI | n | 8868 | 2647 | 4190 | 0.474 | 0.128 |
|  | Mean±SD | 22.9±3.9 | 23.0±3.7 | 22.8±3.7 |  |  |

NPE, never picky eater; PPE, persistent picky eater; Non-PPE, non-persistent picky eater.

Significant differences between the three main categories were calculated using one-way ANOVA.

The table includes only children alive at 1 year.

**Supplementary Table 2** Comparison of food-related behaviours, parent-directed behaviours and mealtimes at 5.5 years between children who were PPE and NPE, and between children who were Non-PPE and NPE (unadjusted analyses)

|  | **Behaviour/perception** | **Answers** | **NPE vs PPE** | | | **NPE vs Non-PPE** | | |
| --- | --- | --- | --- | --- | --- | --- | --- | --- |
|  |  |  | **n** | **OR (95% CI)** | **p value** | **n** | **OR (95% CI)** | **p value** |
| **food behaviours** | Child likes to try different foods | Yes, most times | 938 | 0.13 (0.07, 0.24) | <0.001 | 953 | 0.17 (0.11, 0.26) | <0.001 |
|  |  | Yes, sometimes | 1027 | Ref. |  | 1091 | Ref. |  |
|  |  | Not at all | 510 | 92.83 (64.77, 133.04) | <0.001 | 256 | 24.05 (16.96, 34.1) | <0.001 |
|  | Child seems to enjoy eating | Yes, most times | 1816 | Ref. |  | 1839 | Ref. |  |
|  |  | Yes, sometimes | 535 | 18.02 (14.06, 23.09) | <0.001 | 433 | 10.26 (7.98, 13.19) | <0.001 |
|  |  | Not at all | 128 | 172.91 (85.8, 348.5) | <0.001 | 33 | 29.60 (13.51, 64.81) | <0.001 |
|  | Child plays with food rather than eating eagerly | Yes, most times | 152 | 46.25 (29.63, 72.19) | <0.001 | 73 | 15.77 (9.56, 26.02) | <0.001 |
|  |  | Yes, sometimes | 719 | 7.43 (5.92, 9.34) |  | 608 | 4.39 (3.45, 5.59) |  |
|  |  | Not at all | 1602 | Ref. | <0.001 | 1611 | Ref. | <0.001 |
|  | Child finishes all the food on plate | Yes, most times | 1427 | Ref. |  | 1420 | Ref. |  |
|  |  | Yes, sometimes | 870 | 7.22 (5.66, 9.22) | <0.001 | 774 | 5.37 (4.14, 6.97) | <0.001 |
|  |  | Not at all | 185 | 49.73 (33.03, 74.87) | <0.001 | 108 | 25.41 (16.26, 39.68) | <0.001 |
| **parental directed behaviours** | Child helps to choose food from cupboard/fridge | Yes, often | 644 | 1.36 (1.10, 1.69) | 0.005 | 586 | 1.32 (1.03, 1.70) | 0.030 |
|  |  | Yes, sometimes | 1409 | Ref. |  | 1315 | Ref. |  |
|  |  | Never/rarely | 421 | 0.98 (0.75, 1.28) | 0.888 | 398 | 1.06 (0.78, 1.43) | 0.722 |
|  | Child comes shopping with mother and helps choose food | Yes, often | 651 | 1.26 (1.01, 1.56) | 0.040 | 575 | 0.88 (0.68, 1.15) | 0.362 |
|  |  | Yes, sometimes | 1360 | Ref. |  | 1294 | Ref. |  |
|  |  | Never/rarely | 466 | 1.02 (0.79, 1.32) | 0.876 | 431 | 0.87 (0.64, 1.16) | 0.340 |
|  | Child helps with cooking/food preparation | Yes, often | 226 | 0.76 (0.52, 1.11) | 0.150 | 222 | 1.08 (0.72, 1.62) | 0.720 |
|  |  | Yes, sometimes | 1479 | Ref. |  | 1368 | Ref. |  |
|  |  | Never/rarely | 770 | 1.69 (1.38, 2.07) | <0.001 | 709 | 1.99 (1.57, 2.51) | <0.001 |
|  | Child helps get things for meal/sets table | Yes, often | 822 | 0.63 (0.51, 0.79) | <0.001 | 806 | 0.87 (0.68, 1.11) | 0.25 |
|  |  | Yes, sometimes | 1456 | Ref. |  | 1335 | Ref. |  |
|  |  | Never/rarely | 197 | 2.84 (2.09, 3.85) | <0.001 | 158 | 2.53 (1.76, 3.63) | <0.001 |
|  | Child helps clear up after meals | Yes, often | 577 | 0.65 (0.50, 0.84) | 0.001 | 566 | 0.89 (0.67, 1.19) | 0.432 |
|  |  | Yes, sometimes | 1353 | Ref. |  | 1244 | Ref. |  |
|  |  | Never/rarely | 542 | 1.69 (1.35, 2.11) | <0.001 | 485 | 1.79 (1.39, 2.32) | <0.001 |
|  | Family has proper cooked meal every day | Yes | 2121 | Ref. |  | 2006 | Ref. |  |
|  |  | No | 343 | 2.22 (1.74, 2.84) | <0.001 | 282 | 1.55 (1.14, 2.09) | 0.005 |
| **mealtimes** | Mealtimes are enjoyable for everyone | Mostly/quite often | 1848 | Ref. |  | 1812 | Ref. |  |
|  |  | Occasionally | 572 | 4.81 (3.90, 5.93) | <0.001 | 454 | 3.01 (2.36, 3.83) | <0.001 |
|  |  | Never | 41 | 18.28 (8.86, 37.71) | <0.001 | 16 | 4.09 (1.47, 11.35) | 0.007 |
|  | Mealtimes are a rush | Mostly/quite often | 217 | 1.78 (1.31, 2.42) | <0.001 | 195 | 1.74 (1.22, 2.48) | 0.002 |
|  |  | Occasionally | 1568 | Ref. |  | 1469 | Ref. |  |
|  |  | Never | 658 | 1.16 (0.94, 1.44) | 0.176 | 598 | 1.02 (0.79, 1.32) | 0.883 |
|  | Mealtimes give time to talk to each other | Mostly/quite often | 1771 | Ref. |  | 1692 | Ref. |  |
|  |  | Occasionally | 604 | 2.30 (1.87, 2.84) | <0.001 | 525 | 1.87 (1.46, 2.39) | <0.001 |
|  |  | Never | 78 | 4.59 (2.90, 7.27) | <0.001 | 57 | 2.82 (1.59, 5.01) | <0.001 |
|  | Mealtimes include arguments between the children | Mostly/quite often | 366 | 1.97 (1.53, 2.54) | <0.001 | 323 | 1.84 (1.37, 2.47) | <0.001 |
|  |  | Occasionally | 1302 | Ref. |  | 1221 | Ref. |  |
|  |  | Never | 768 | 1.01 (0.81, 1.26) | 0.925 | 719 | 1.00 (0.78, 1.29) | 0.981 |
|  | Mealtimes include arguments between adults & children | Mostly/quite often | 162 | 2.33 (1.66, 3.26) | <0.001 | 133 | 2.07 (1.39, 3.07) | <0.001 |
|  |  | Occasionally | 1202 | Ref. |  | 1101 | Ref. |  |
|  |  | Never | 1081 | 0.61 (0.50, 0.75) | <0.001 | 1031 | 0.67 (0.53, 0.85) | 0.001 |

NPE, never picky eater; PPE, persistent picky eater; Non-PPE, non-persistent picky eater.

The picky eating reference category is always NPE in both comparisons. The exposure reference is presented as ‘Ref.’.

**Supplementary Table 3** Feeding behaviours at school age in children who were PPE or Non-PPE compared with those who were NPE during the first 5 years of life (unadjusted analyses)

| **Age** | Questions:  **During the past year the child…** | **Reply** | **PPE vs NPE** | | | **Non-PPE vs NPE** | | |
| --- | --- | --- | --- | --- | --- | --- | --- | --- |
|  |  |  | **n** | **OR (95% CI)** | **p value** | **n** | **OR (95% CI)** | **p value** |
| **5.5 years** | indicated difficulties in eating what the mother wanted | No | 1467 |  |  | 1507 |  |  |
|  |  | Yes | 1016 | 81.43 (51.5, 128.76) | <0.001 | 802 | 16.35 (12.18, 21.94) | <0.001 |
|  | has deliberately not eaten sufficient amount of food | No | 2125 |  |  | 2033 |  |  |
|  |  | Yes | 344 | 5.68 (4.46, 7.22) | <0.001 | 265 | 4.37 (3.31, 5.76) | <0.001 |
|  | has refused to eat the offered food | No | 1359 |  |  | 1400 |  |  |
|  |  | Yes | 1115 | 43.22 (29.2, 63.98) | <0.001 | 898 | 10.37 (7.85, 13.7) | <0.001 |
|  | has been choosy with food | No | 1080 |  |  | 1098 |  |  |
|  |  | Yes | 1400 | 141.64 (58.45, 343.2) | <0.001 | 1205 | 19.99 (12.99, 30.76) | <0.001 |
|  | has over-eaten | No | 2043 |  |  | 1850 |  |  |
|  |  | Yes | 436 | 0.2 (0.14, 0.3) | <0.001 | 451 | 0.48 (0.35, 0.67) | <0.001 |
|  | was difficult to get into eating routine | No | 2190 |  |  | 2127 |  |  |
|  |  | Yes | 275 | 21.65 (15.79, 29.69) | <0.001 | 163 | 13.03 (9.21, 18.44) | <0.001 |
|  |  |  |  |  |  |  |  |  |
| **7 years** | indicated difficulties in eating what the mother wanted | No | 1356 |  |  | 1395 |  |  |
|  |  | Yes | 900 | 34.85 (25.11, 48.36) | <0.001 | 685 | 10.28 (7.85, 13.47) | <0.001 |
|  | has deliberately not eaten sufficient amount of food | No | 1944 |  |  | 1830 |  |  |
|  |  | Yes | 335 | 4.32 (3.39, 5.51) | <0.001 | 271 | 3.88 (2.94, 5.13) | <0.001 |
|  | has refused to eat the offered food | No | 1254 |  |  | 1278 |  |  |
|  |  | Yes | 1025 | 16.14 (12.18, 21.38) | <0.001 | 823 | 6.68 (5.14, 8.68) | <0.001 |
|  | has been choosy with food | No | 1041 |  |  | 1056 |  |  |
|  |  | Yes | 1238 | 53.3 (31.08, 91.41) | <0.001 | 1045 | 16.2 (10.95, 23.96) | <0.001 |
|  | has over-eaten | No | 1856 |  |  | 1688 |  |  |
|  |  | Yes | 423 | 0.39 (0.28, 0.53) | <0.001 | 413 | 0.48 (0.34, 0.68) | <0.001 |
|  | was difficult to get into eating routine | No | 2056 |  |  | 1979 |  |  |
|  |  | Yes | 223 | 13.31 (9.66, 18.36) | <0.001 | 122 | 6.59 (4.52, 9.6) | <0.001 |
|  |  |  |  |  |  |  |  |  |
| **8.5 years** | indicated difficulties in eating what the mother wanted | No | 1321 |  |  | 1360 |  |  |
|  |  | Yes | 735 | 21.53 (16.29, 28.44) | <0.001 | 542 | 7.34 (5.66, 9.52) | <0.001 |
|  | has deliberately not eaten sufficient amount of food | No | 1831 |  |  | 1722 |  |  |
|  |  | Yes | 213 | 4.47 (3.34, 5.99) | <0.001 | 165 | 3.65 (2.59, 5.12) | <0.001 |
|  | has refused to eat the offered food | No | 1277 |  |  | 1299 |  |  |
|  |  | Yes | 767 | 13.98 (10.79, 18.1) | <0.001 | 590 | 6.11 (4.72, 7.9) | <0.001 |
|  | has been choosy with food | No | 974 |  |  | 999 |  |  |
|  |  | Yes | 1078 | 31.84 (20.52, 49.43) | <0.001 | 895 | 8.94 (6.45, 12.38) | <0.001 |
|  | has over-eaten | No | 1654 |  |  | 1505 |  |  |
|  |  | Yes | 388 | 0.48 (0.36, 0.65) | <0.001 | 381 | 0.65 (0.47, 0.9) | 0.011 |
|  | was difficult to get into eating routine | No | 1885 |  |  | 1806 |  |  |
|  |  | Yes | 152 | 13.21 (8.96, 19.47) | <0.001 | 80 | 6.33 (4.01, 10.01) | <0.001 |

NPE, never picky eater; PPE, persistent picky eater; Non-PPE, non-persistent picky eater.

The picky eating reference category is always NPE in both comparisons. The empty cells in the OR (95% CI) and p value represent the reference categories (‘No’).

**Supplementary Table 4**Feeding behaviours at school age in children who were PPE or Non-PPE compared with those who were NPE during the first 5 years of life (adjusted for early maternal worry only)

| **Age** | **Questions: During the past year the child…** | **Predictor variable** | **PPE vs NPE** | | **Non-PPE vs NPE** | |
| --- | --- | --- | --- | --- | --- | --- |
|  |  |  | **OR (95% CI)** | **p value** | **OR (95% CI)** | **p value** |
| **5.5 years** | indicated difficulties in eating what the mother wanted | Picky | 69.28 (43.27, 110.94) | <0.001 | 14.45 (10.48, 19.91) | <0.001 |
|  |  | Maternal worry | 1.6 (1.07, 2.38) | 0.021 | 1.45 (1.02, 2.06) | 0.040 |
|  | has deliberately not eaten sufficient amount of food | Picky | 4.99 (3.8, 6.57) | <0.001 | 4.25 (3.08, 5.86) | <0.001 |
|  |  | Maternal worry | 1.43 (1.04, 1.96) | 0.027 | 1.1 (0.75, 1.62) | 0.624 |
|  | has refused to eat the offered food | Picky | 37.54 (24.87, 56.68) | <0.001 | 9.49 (6.98, 12.9) | <0.001 |
|  |  | Maternal worry | 1.62 (1.11, 2.36) | 0.012 | 1.23 (0.88, 1.73) | 0.223 |
|  | has been choosy with food | Picky | 124.02 (50.78, 302.91) | <0.001 | 18.73 (11.84, 29.65) | <0.001 |
|  |  | Maternal worry | 1.43 (0.96, 2.15) | 0.08 | 1.25 (0.86, 1.82) | 0.238 |
|  | has over-eaten | Picky | 0.19 (0.13, 0.3) | <0.001 | 0.43 (0.3, 0.63) | <0.001 |
|  |  | Maternal worry | 1.17 (0.78, 1.76) | 0.443 | 1.35 (0.93, 1.98) | 0.117 |
|  | was difficult to get into eating routine | Picky | 18.34 (13.03, 25.82) | <0.001 | 12.31 (8.33, 18.19) | <0.001 |
|  |  | Maternal worry | 1.4 (1.01, 1.96) | 0.044 | 1.05 (0.68, 1.61) | 0.824 |
|  | indicated difficulties in eating what the mother wanted | Picky | 26 (18.56, 36.43) | <0.001 | 8.32 (6.19, 11.19) | <0.001 |
|  |  | Maternal worry | 2.61 (1.78, 3.82) | <0.001 | 1.83 (1.3, 2.59) | 0.001 |
| **7 years** | has deliberately not eaten sufficient amount of food | Picky | 4.39 (3.34, 5.78) | <0.001 | 3.94 (2.86, 5.45) | <0.001 |
|  |  | Maternal worry | 1.03 (0.74, 1.44) | 0.848 | 1 (0.68, 1.49) | 0.989 |
|  | has refused to eat the offered food | Picky | 14.75 (10.86, 20.05) | <0.001 | 6.28 (4.68, 8.42) | <0.001 |
|  |  | Maternal worry | 1.32 (0.93, 1.87) | 0.121 | 1.11 (0.8, 1.56) | 0.531 |
|  | has been choosy with food | Picky | 47.15 (27.11, 82.02) | <0.001 | 15.12 (9.96, 22.95) | <0.001 |
|  |  | Maternal worry | 1.35 (0.9, 2.03) | 0.145 | 1.13 (0.77, 1.65) | 0.539 |
|  | has over-eaten | Picky | 0.39 (0.27, 0.55) | <0.001 | 0.48 (0.33, 0.71) | <0.001 |
|  |  | Maternal worry | 1.04 (0.7, 1.54) | 0.846 | 1.07 (0.71, 1.61) | 0.744 |
|  | was difficult to get into eating routine | Picky | 10.92 (7.69, 15.51) | <0.001 | 7.39 (4.85, 11.27) | <0.001 |
|  |  | Maternal worry | 1.5 (1.06, 2.14) | 0.023 | 0.66 (0.39, 1.11) | 0.115 |
| **8 years** | indicated difficulties in eating what the mother wanted | Picky | 17.5 (13, 23.55) | <0.001 | 6.82 (5.07, 9.18) | <0.001 |
|  |  | Maternal worry | 1.87 (1.29, 2.7) | 0.001 | 1.18 (0.83, 1.68) | 0.366 |
|  | has deliberately not eaten sufficient amount of food | Picky | 4.37 (3.14, 6.09) | <0.001 | 3.76 (2.53, 5.58) | <0.001 |
|  |  | Maternal worry | 1.06 (0.72, 1.57) | 0.763 | 0.85 (0.52, 1.39) | 0.525 |
|  | has refused to eat the offered food | Picky | 12.4 (9.34, 16.46) | <0.001 | 5.93 (4.41, 7.97) | <0.001 |
|  |  | Maternal worry | 1.32 (0.94, 1.88) | 0.112 | 1.09 (0.77, 1.55) | 0.622 |
|  | has been choosy with food | Picky | 25.96 (16.51, 40.81) | <0.001 | 8.1 (5.67, 11.59) | <0.001 |
|  |  | Maternal worry | 1.86 (1.23, 2.81) | 0.003 | 1.36 (0.93, 1.98) | 0.108 |
|  | has over-eaten | Picky | 0.46 (0.33, 0.65) | <0.001 | 0.64 (0.44, 0.93) | 0.019 |
|  |  | Maternal worry | 1.17 (0.79, 1.73) | 0.431 | 1.19 (0.79, 1.78) | 0.409 |
|  | was difficult to get into eating routine | Picky | 13.64 (8.95, 20.79) | <0.001 | 6.7 (3.96, 11.32) | <0.001 |
|  |  | Maternal worry | 0.94 (0.62, 1.42) | 0.777 | 0.65 (0.34, 1.25) | 0.195 |

NPE, never picky eater; PPE, persistent picky eater; Non-PPE, non-persistent picky eater.

The picky eating reference category is always NPE in both comparisons. The empty cells in the OR (95% CI) and p value represent the reference categories (‘No’).

**Supplementary Table 5**Moderating effect of early maternal worry about her child being choosy with food at aged 15 months on feeding behaviours during early school years in children who were PPE or Non-PPE compared with those who were NPE during the first 5 years of life (adjusted analyses)

| **Age** | **Questions:**  **During the past year the child…** | **Predictor variable** | **PPE vs NPE** | | **Non-PPE vs NPE** | | |
| --- | --- | --- | --- | --- | --- | --- | --- |
|  |  |  | **OR (95% CI)** | **p value** | **OR (95% CI)** | **p value** |  |
| **5.5 years** | indicated difficulties in eating what the mother wanted | Picky | 70.91 (39.31, 127.93) | <0.001 | 17.08 (11.21, 26.03) | <0.001 |  |
|  |  | Maternal worry | 1.84 (1.17, 2.89) | 0.009 | 1.85 (1.17, 2.9) | 0.008 |  |
|  |  | Picky x Maternal worry | 0.8 (0.27, 2.41) | 0.697^a^ | 0.55 (0.26, 1.18) | 0.127 ^a^ |  |
|  | has deliberately not eaten sufficient amount of food | Picky | 5.03 (3.66, 6.93) | <0.001 | 4.25 (2.89, 6.25) | <0.001 |  |
|  |  | Maternal worry | 1.60 (0.8, 3.19) | 0.183 | 1.56 (0.78, 3.11) | 0.207 |  |
|  |  | Picky x Maternal worry | 0.96 (0.44, 2.12) | 0.922 ^a^ | 0.68 (0.29, 1.59) | 0.369 |  |
|  | has refused to eat the offered food | Picky | 39.31 (23.45, 65.91) | <0.001 | 12.03 (7.98, 18.12) | <0.001 |  |
|  |  | Maternal worry | 1.5 (0.96, 2.37) | 0.078 | 1.51 (0.96, 2.37) | 0.077 |  |
|  |  | Picky x Maternal worry | 1.02 (0.38, 2.71) | 0.972 ^a^ | 0.52 (0.25, 1.08) | 0.078 |  |
|  | has been choosy with food | Picky | 123.37 (39.37, 386.6) | <0.001 | 21.55 (11.29, 41.13) | <0.001 |  |
|  |  | Maternal worry | 1.36 (0.88, 2.12) | 0.166 | 1.37 (0.88, 2.12) | 0.161 |  |
|  |  | Picky x Maternal worry | 0.77 (0.12, 4.89) | 0.781 | 0.52 (0.19, 1.39) | 0.189 |  |
|  | has over-eaten | Picky | 0.28 (0.18, 0.46) | <0.001 | 0.48 (0.29, 0.77) | 0.003 |  |
|  |  | Maternal worry | 1.52 (0.93, 2.49) | 0.096 | 1.53 (0.93, 2.51) | 0.091 |  |
|  |  | Picky x Maternal worry | 0.32 (0.12, 0.88) | 0.028 | 0.57 (0.24, 1.38) | 0.213 |  |
|  | was difficult to get into eating routine | Picky | 21.75 (14.63, 32.35) | <0.001 | 15.89 (10.09, 25.03) | <0.001 |  |
|  |  | Maternal worry | 2.9 (1.19, 7.07) | 0.019 | 2.88 (1.18, 7.02) | 0.02 |  |
|  |  | Picky x Maternal worry | 0.45 (0.17, 1.18) | 0.104 ^a^ | 0.27 (0.10, 0.75) | 0.012 |  |
| **7 years** | indicated difficulties in eating what the mother wanted | Picky | 30.48 (20.09, 46.24) | <0.001 | 11.66 (7.91, 17.18) | <0.001 |  |
|  |  | Maternal worry | 3.33 (2.07, 5.35) | <0.001 | 3.35 (2.09, 5.38) | <0.001 |  |
|  |  | Picky x Maternal worry | 0.6 (0.25, 1.46) | 0.262 | 0.27 (0.13, 0.56) | <0.001 ^a^ |  |
|  | has deliberately not eaten sufficient amount of food | Picky | 4.16 (3.06, 5.67) | <0.001 | 3.35 (2.29, 4.92) | <0.001 |  |
|  |  | Maternal worry | 0.63 (0.25, 1.6) | 0.334 | 0.64 (0.25, 1.61) | 0.342 |  |
|  |  | Picky x Maternal worry | 1.79 (0.65, 4.88) | 0.258 | 1.73 (0.60, 4.94) | 0.309 |  |
|  | has refused to eat the offered food | Picky | 14.92 (10.28, 21.66) | <0.001 | 6.7 (4.63, 9.68) | <0.001 |  |
|  |  | Maternal worry | 1.37 (0.85, 2.22) | 0.194 | 1.38 (0.85, 2.23) | 0.187 |  |
|  |  | Picky x Maternal worry | 0.85 (0.4, 1.78) | 0.664 | 0.66 (0.33, 1.34) | 0.254 |  |
|  | has been choosy with food | Picky | 66.76 (29.51, 151.02) | <0.001 | 23.49 (12.28, 44.92) | <0.001 |  |
|  |  | Maternal worry | 1.51 (0.95, 2.41) | 0.081 | 1.52 (0.95, 2.42) | 0.079 |  |
|  |  | Picky x Maternal worry | 0.39 (0.12, 1.3) | 0.126 | 0.32 (0.12, 0.82) | 0.018 |  |
|  | has over-eaten | Picky | 0.43 (0.29, 0.64) | <0.001 | 0.39 (0.23, 0.66) | <0.001 |  |
|  |  | Maternal worry | 1.15 (0.66, 1.99) | 0.620 | 1.15 (0.66, 1.99) | 0.623 |  |
|  |  | Picky x Maternal worry | 0.81 (0.35, 1.84) | 0.611 | 1.07 (0.43, 2.68) | 0.886 |  |
|  | was difficult to get into eating routine | Picky | 11.78 (7.91, 17.54) | <0.001 | 8.27 (5.12, 13.37) | <0.001 |  |
|  |  | Maternal worry | 1.27 (0.38, 4.19) | 0.697 | 1.26 (0.38, 4.15) | 0.706 |  |
|  |  | Picky x Maternal worry | 1.35 (0.38, 4.75) | 0.643 ^a^ | 0.49 (0.13, 1.86) | 0.293 |  |
| **8.5 years** | indicated difficulties in eating what the mother wanted | Picky | 19.03 (13.28, 27.26) | <0.001 | 9.7 (6.65, 14.16) | <0.001 |  |
|  |  | Maternal worry | 2.01 (1.19, 3.38) | 0.009 | 2.03 (1.21, 3.42) | 0.008 |  |
|  |  | Picky x Maternal worry | 0.9 (0.41, 1.94) | 0.779 ^a^ | 0.33 (0.16, 0.68) | 0.003 |  |
|  | has deliberately not eaten sufficient amount of food | Picky | 4.33 (2.98, 6.3) | <0.001 | 3.99 (2.55, 6.24) | <0.001 |  |
|  |  | Maternal worry | 1.12 (0.44, 2.86) | 0.817 | 1.1 (0.43, 2.82) | 0.836 |  |
|  |  | Picky x Maternal worry | 0.97 (0.34, 2.75) | 0.950 | 0.7 (0.23, 2.13) | 0.529 |  |
|  | has refused to eat the offered food | Picky | 11.67 (8.37, 16.28) | <0.001 | 6.44 (4.47, 9.28) | <0.001 |  |
|  |  | Maternal worry | 1.23 (0.72, 2.13) | 0.448 | 1.25 (0.72, 2.16) | 0.426 |  |
|  |  | Picky x Maternal worry | 1.32 (0.63, 2.79) | 0.460 | 0.84 (0.4, 1.77) | 0.655 |  |
|  | has been choosy with food | Picky | 22.97 (13.64, 38.67) | <0.001 | 9.79 (6.07, 15.77) | <0.001 |  |
|  |  | Maternal worry | 1.71 (1.05, 2.77) | 0.031 | 1.72 (1.06, 2.79) | 0.029 |  |
|  |  | Picky x Maternal worry | 1.45 (0.47, 4.52) | 0.517 ^a^ | 0.53 (0.23, 1.2) | 0.127 |  |
|  | has over-eaten | Picky | 0.54 (0.36, 0.82) | 0.004 | 0.73 (0.46, 1.17) | 0.192 |  |
|  |  | Maternal worry | 1.24 (0.7, 2.2) | 0.459 | 1.24 (0.70, 2.19) | 0.465 |  |
|  |  | Picky x Maternal worry | 0.74 (0.32, 1.71) | 0.487 | 0.78 (0.32, 1.87) | 0.577 |  |
|  | was difficult to get into eating routine | Picky | 13.08 (8.17, 20.93) | <0.001 | 6.42 (3.49, 11.81) | <0.001 |  |
|  |  | Maternal worry | 1.11 (0.70, 1.76) | 0.657 | 0.82 (0.38, 1.73) | 0.595 |  |
|  |  | Picky x Maternal worry | - | - | - | - |  |

NPE, never picky eater; PPE, persistent picky eater; Non-PPE, non-persistent picky eater.

NPE is always the reference category for the predictor (being a picky eater). The answer ‘Not worried’ for the moderator is always the reference category (early maternal worry when her child was aged 15 months).

All analyses were adjusted for maternal educational attainment, pre-pregnancy BMI, and child’s sex, parity and birthweight.

The interaction term is presented as the predictor x moderator.

All models were significant (p<0.005). All the unadjusted models were significant (predictor and moderator only) (data not shown).

^a^Both components of the interaction term (predictor and moderator) were significant in their corresponding adjusted models.

Cells represented as (-) had extremely high odds ratios (not shown).

**Supplementary Table 6** Effect of maternal strategies for feeding her child at age 5.5 years on the presence of feeding difficulties at age 6.5 and 8.5 years in children who were PPE or Non-PPE combined (unadjusted analyses)

| **Maternal strategies to improve feeding behaviour when she was faced with feeding difficulties when the child was aged 5.5 years** | | **Is the child difficult to feed? (Reference: No)** | | | | | |
| --- | --- | --- | --- | --- | --- | --- | --- |
|  |  | **Age 6.5 years** | | | **Age 8.5 years** | | |
|  |  | **n** | **OR (95% CI)** | **p value** | **n** | **OR (95% CI)** | **p value** |
| Mother lets the child eat something else | No | 245 |  |  | 227 |  |  |
|  | Yes | 617 | 1.63 (1.09, 2.43) | 0.018 | 561 | 1.80 (1.26, 2.56) | 0.001 |
| Mother encourages child to eat it the food by making up a game or story | No | 444 |  |  | 408 |  |  |
|  | Yes | 420 | 2.09 (1.39, 3.13) | <0.001 | 381 | 1.67 (1.18, 2.35) | 0.004 |
| Mother mixes food with other food that the child likes and will eat | No | 558 |  |  | 511 |  |  |
|  | Yes | 308 | 1.14 (0.76, 1.71) | 0.535 | 281 | 1.21 (0.84, 1.73) | 0.304 |
| Mother lets child put sauce on food to cover up the taste/appearance | No | 428 |  |  | 383 |  |  |
|  | Yes | 435 | 1.42 (0.96, 2.09) | 0.079 | 406 | 1.37 (0.97, 1.91) | 0.071 |
| Mother tries to persuade the child to eat a very small amount | No | 32 |  |  | 30 |  |  |
|  | Yes | 837 | 2.53 (1.14, 5.61) | 0.022 | 764 | 2.48 (1.17, 5.25) | 0.018 |
| Mother does not let child to leave table/have anything else until it finishes its plate | No | 593 |  |  | 544 |  |  |
|  | Yes | 272 | 1.20 (0.79, 1.84) | 0.393 | 247 | 0.98 (0.68, 1.40) | 0.902 |
| Mother tries to encourage child with rewards if it finishes the food | No | 434 |  |  | 405 |  |  |
|  | Yes | 435 | 1.51 (1.02, 2.22) | 0.039 | 389 | 1.53 (1.09, 2.15) | 0.015 |
| Mother takes the food away and gives something else to eat | No | 387 |  |  | 354 |  |  |
|  | No | 479 | 1.41 (0.96, 2.08) | 0.081 | 437 | 1.24 (0.88, 1.73) | 0.219 |
| Mother does not make issue of child not eating the food | Yes | 52 |  |  | 43 |  |  |
|  | No | 814 | 1.72 (0.86, 3.44) | 0.127 | 746 | 0.94 (0.44, 2.00) | 0.871 |
| Mother tries same food again on a different day | No | 151 |  |  | 140 |  |  |
|  | Yes | 714 | 1.29 (0.80, 2.09) | 0.295 | 651 | 1.54 (1.02, 2.33) | 0.039 |

NPE, never picky eater; PPE, persistent picky eater; Non-PPE, non-persistent picky eater.

Reference category in the strategy and facing feeding difficulties is always ‘No’.

Three children who were PPE and three who were Non-PPE, whose mothers replied with “Never difficult” were excluded from the analysis.
